# How do recurrent malaria infections occur in clinical cohorts: a mathematical modelling study to support study planning

**DOI:** 10.1186/s12936-025-05594-1

**Published:** 2025-10-13

**Authors:** Ralf Krumkamp, Lydia Helen Rautman, Oumou Maiga-Ascofaré, Jürgen May, Eva Lorenz

**Affiliations:** 1 Department of Infectious Disease Epidemiology, Bernhard Nocht Institute for Tropical Medicine, Bernhard Nocht Str. 74, 20359 Hamburg, Germany; 2 German Center for Infection Research, Hamburg-Borstel-Lübeck-Riems, Germany; 3 Kumasi Centre for Collaborative Research in Tropical Medicine, Kumasi, Ghana; 4 Department of Tropical Medicine I, University Medical Center Hamburg-Eppendorf (UKE), Hamburg, Germany

**Keywords:** Cohort studies, Sample size, Mathematical modelling, Malaria, Recurrent infection

## Abstract

**Background:**

Recurrent events of infectious diseases are common and the subject of analyses in many clinical studies. A proper understanding of disease occurrence over time within a cohort provides a basis for study planning and sample size estimation. This study mathematically describes the recurrence of malaria in a malaria-naïve cohort and highlights the necessary assumptions to inform study planning.

**Methods:**

To represent different disease transmission scenarios, five mathematical models with different levels of complexity were constructed to mimic possible real-life scenarios. Model A represents the simplest model with constant infection risk, Model B includes protection due to treatment and reduced individual susceptibility after each infection, Model C shows preventive effects from a vaccination, Model D explores heterogeneous transmission with varying levels of infection risks, and Model E captures temporal dynamics through seasonal variation in infection risk. The models were implemented as compartmental models using a system of ordinary differential equations.

**Results:**

The different transmission scenarios strongly affected the pattern of recurrent infections. Models A and B had the same number of cases with infections; however, due to treatment effects and immunity development, the number of recurrent events was lower in Model B. Compared to Model B, Model C showed a substantial reduction in both first and recurring infections. In Model D, the subpopulation with a high transmission risk had a higher proportion of recurrent infections, with nearly 100% of this group experiencing more than one infection. Model E demonstrated how seasonal transmission risk leads to temporal dynamics with strong fluctuations in the occurrence of infections. Based on these models, we provide examples of how final cohort sizes can be estimated for different transmission settings.

**Conclusions:**

Recurrent infections in longitudinal studies cannot be estimated directly from disease frequency data. However, this study provides a simple set of equations to calculate the number of expected recurrent events. These models can be easily adapted to represent additional transmission and infection dynamics or to model other recurrent diseases like influenza.

**Supplementary Information:**

The online version contains supplementary material available at 10.1186/s12936-025-05594-1.

## Background

In many longitudinal cohort studies, single events per person, like time to disease onset or death, are analysed and survival analysis is used to study intervention effects. For this kind of data Kaplan–Meier methods to estimate the survival function and Cox proportional hazard models to estimate the hazard ratio are common analytical methods [1]. However, several diseases cause outcomes that can recur within one patient. Epileptic seizures, asthma attacks or cardiac arrythmia are examples of non-communicable diseases that may recur. Recurrent events are also common in communicable diseases. Infectious diseases with short recovery times and partial or fast-waning immunity can cause several episodes per person. Examples include clinical malaria episodes due to *Plasmodium falciparum* parasite infection and influenza infections during endemic seasons. Recurrent disease episodes in longitudinal studies are of clinical interest as they provide information about a patient’s prognosis, individual susceptibility or differences in infection risk. For example, in malaria vaccine efficacy trials (phase III), the time to first disease episode after the primary series of vaccinations is often the main endpoint. However, the efficacy of a vaccine against recurrent infections is also reported [2, 3]. Various methods have been proposed for analysing the effects of interventions on data from recurrent events in longitudinal studies, such as extensions of the Cox model (Andersen-Gill, Prentice-Williams-Peterson, Wei-Lin-Weissfeld models) and frailty models [4]. Sample size formulas for the analyses of recurrent events have been developed and are well described [5–9]. These calculations determine the number of participants needed in an intervention and control group to estimate an effect with a desired level of precision.

Limited methodological guidance exists about how recurrent disease episodes are represented in longitudinal studies. However, a proper understanding is relevant for study planning and conduct; for example, to make assumptions for sample size estimation or predict recurrent disease patterns in a cohort to plan follow-up procedures. It is important to note that the occurrence of recurrent infections cannot be predicted from disease frequency data directly. Recurrence is a temporal process in which individuals transition towards states of experiencing successive disease events [10]. This study aims to demonstrate the dynamics of disease recurrence in population cohorts and is structured as follows: (1) introduction of a mathematical framework for modelling recurrent malaria infections, (2) application of these models to explore different patterns of disease recurrence using real-life scenarios, and (3) use of the models to estimate the number of cases and events in longitudinal studies. Although malaria is used as an example, the principles and methods presented here can be applied to other, also non-communicable conditions with recurrent outcomes.

## Methods

A common estimator for the frequency of disease in a population at risk is the incidence proportion (IP), also called cumulative incidence. The IP ranges from 0 to 100%, and shows the individual risk to experience an outcome over a given time period. Multiple events per person are not considered. The incidence rate (IR) or event rate, however, represents the number of disease episodes within a population over a defined period of time. The IR can exceed the value of 1, which indicates that at least some individuals experienced recurrent outcomes [11, 12]. IP and IR are mathematically related. Assuming recurrent events occur independently within a closed population, the proportion of individuals who experience at least one event over a given period of time is given by IP = 1-exp(-IR∙time) [11, 13], where time is measured in the same unit as the IR. IR is the parameter used to define the frequency of recurrent events in the modelled populations.

A system of ordinary differential equations was used to calculate compartmental models, where individuals move through stages of no infection, a single infection, up to *k* recurrent infections. Five mathematical models (A–E) with different levels of complexity were constructed to calculate the expected number of recurrent malaria infections.

In short, Model A represents the simplest model with independent, uniform infection risk, Model B includes the effect of decreasing individual susceptibility after each infection, Model C shows the impact of reduced infection risk due to vaccination, Model D illustrates the effect of heterogeneous clustered infection risks, and Model E includes seasonal variation in infection risk. The number of recurrent events by time *t*, over a timespan of 2 years, was modelled. Each model starts with malaria-naïve, fully susceptible individuals and partial immunity due to maternal antibodies is not considered. The occurrence of malaria infections is modeled and no further clinical differentiation, like severe malaria or asymptomatic infection, is made. Models start at *t*_0_ with a value 1 for the susceptible compartment and all other compartments have a value of 0, thus moving proportions over time through compartments are calculated.

**Fig. 1:**
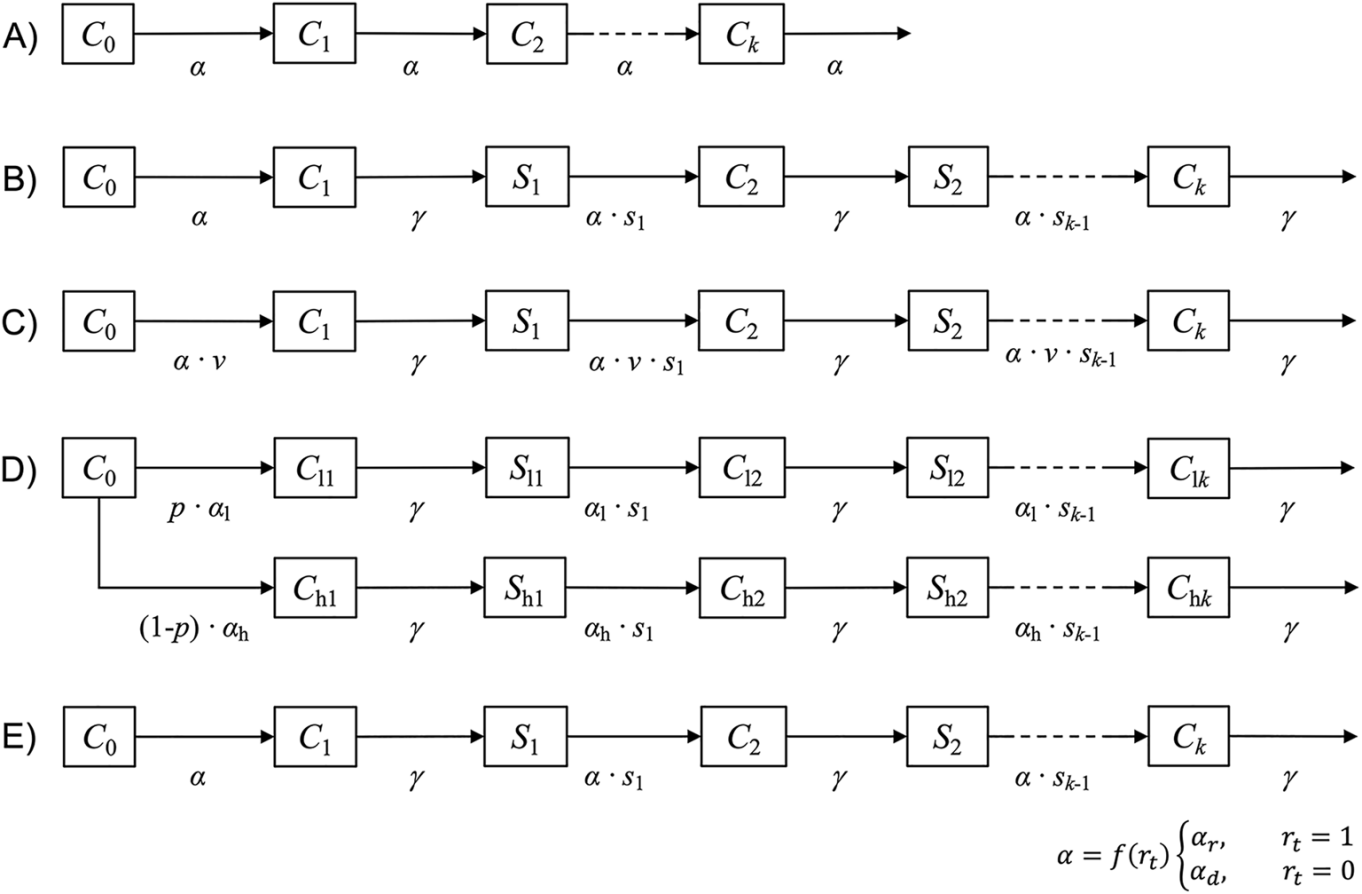
Compartment models on recurrent infections in initially disease-free individuals (C_0_); **A** equal infection risk (α) and individuals are fully susceptible throughout the modelling period while they experience 1 to *k* recurrent infections; **B** individuals are protected for a transit time of *γ* from infection due to ongoing treatment, moving to compartment S_*k*_ where they can become infected again. Previous infections reduce susceptibility by the proportion of s_*k*_; **C** as Model B, but with the addition of the protective effect due to vaccination (*v*); **D** as Model B but with a fraction of the population having a high (*p*) or low (1-*p*) infection rate; **E** as Model B but seasonal infection risk is calculated and *α* changing by values of *r*

In the simplest model (A), individuals are free of infection at *t*_0_, and everyone is fully susceptible throughout the modelling period, i.e., events within subjects are considered independent and occur stochastically. The daily infection rate α is applied in the calculations, which equals the IR over a person-year divided by 365. Disease-free individuals (*C*_0_) get infected at rate *α* and move to compartment *C*_1_ after experiencing a first infection (*k* = 1). This group can be infected a second time (*k* = 2) according to the risk defined by *α* and move to *C*_2_ from where they can become infected again and transition through *k* recurrent infection compartments. The compartmental structure of this model is depicted in Fig. 1a and described in Eqs. 1–3. This simplest model, which has stochastic infection occurrence and a constant infection rate, can be described using the Poisson distribution function given in Eq. 4 [10]. The proportion of *k* infections at time point *t* can be calculated using this equation. However, such a general formula cannot be used to calculate results for the more complex models described below. Figure 1b shows the compartmental structure and Eqs. 5–9 describe the model where infected individuals are protected from reinfection due to ongoing treatment, and each experienced infection reduces susceptibility to recurrent infection, similar to the circle of malaria infections (Model B). Again, the model begins with susceptible individuals (*C*_0_), and first infections occur at rate *α*. Individuals infected for the first time move to compartment *C*_1_, where they are protected from reinfection for a period defined by *γ*. They then transition to the compartment of susceptible individuals with a first infection (*S*_1_). This group has lower susceptibility, which reduces the infection risk *α* by the proportion defined by *s*_1_. Individuals infected a second time are protected due to treatment for the period defined by *γ* and have further reduced susceptibility to reinfection defined by *s*_2_. Further recurrent infections reduce susceptibility, as defined by *s*_k_. Model C has a similar structure as Model B, but vaccination reduces the individual malaria risk for each individual in the population. Thus, the infection risk (α) is multiplied by the proportion of infections still expected in a vaccinated population (*v*), which equals 1 minus vaccine efficacy (Fig. 1c and Eqs. 10–14). The next model extension is the effect of heterogeneous or clustered infection risk, calculated in Model D (Fig. 1d and Eqs. 15–23). The model follows the structure of model B, but the infection risk differs for a fraction of the modelled cohort. The proportion *p* of *C*_0_ has a low infection risk (*α*_l_) while the rest of the cohort (1-*p*) is exposed to high infection risk (*α*_h_). Recurrent infections are modelled separately for the high and low risk groups, and numbers in the respective infection compartments must be summed (*C*_*lk*_ + *C*_*hk*_) to obtain the total number of infections. Model E (Fig. 1e and Eqs. 24–28) follows the structure of Model B and captures seasonal transmission risk. The risk of infection *α* depends on *r*_t_ varying over time, defining a high (*r*_t_ = 1) or low (*r*_t_ = 0) transmission season. During periods of high transmission, the rainy season transmission rate (*α*_r_) becomes active, as does the dry season transmission rate (*α*_d_) during periods of low transmission.

1 $$\frac{{dC_{0} }}{dt} = - {\upalpha } \cdot C_{0}$$

2 $$\frac{{dC_{1} }}{dt} = {\upalpha } \cdot C_{0} - {\upalpha } \cdot C_{1}$$

3 $$\frac{{dC_{k} }}{dt} = {\upalpha } \cdot C_{{{\text{k}} - 1}} - {\upalpha } \cdot C_{k}$$

4 $$C_{k} \left( t \right) = \frac{{\left( {\alpha \cdot t} \right)^{k} }}{k!} \cdot e^{ - \alpha \cdot t} {\text{ for }}k = 0, 1, 2, \cdots$$

5 $$\frac{{dC_{0} }}{dt} = - {\upalpha } \cdot C_{0}$$

6 $$\frac{{dC_{1} }}{dt} = {\upalpha } \cdot C_{0} - \gamma \cdot C_{1}$$

7 $$\frac{{dS_{1} }}{dt} = \gamma \cdot C_{1} - {\upalpha } \cdot s_{1} \cdot S_{1}$$

8 $$\frac{{dC_{k} }}{dt} = {\upalpha } \cdot s_{k - 1} \cdot S_{k - 1} - \gamma \cdot C_{k}$$

9 $$\frac{{dS_{k} }}{dt} = \gamma \cdot C_{k} - {\upalpha } \cdot s_{k} \cdot S_{k}$$

10 $$\frac{{dC_{0} }}{dt} = - {\upalpha } \cdot {\text{v}} \cdot C_{0}$$

11 $$\frac{{dC_{1} }}{dt} = {\upalpha } \cdot {\text{v}} \cdot C_{0} - \gamma \cdot C_{1}$$

12 $$\frac{{dS_{1} }}{dt} = \gamma \cdot C_{1} - {\upalpha } \cdot {\text{v}} \cdot s_{1} \cdot S_{1}$$

13 $$\frac{{dC_{k} }}{dt} = {\upalpha } \cdot {\text{v}} \cdot s_{k - 1} \cdot S_{k - 1} - \gamma \cdot C_{k}$$

14 $$\frac{{dS_{k} }}{dt} = \gamma \cdot C_{k} - {\upalpha } \cdot {\text{v}} \cdot s_{k} \cdot S_{k}$$

15 $$\frac{{dC_{0} }}{dt} = - {\text{p}} \cdot {\upalpha }_{l} \cdot C_{0} - \left( {1 - {\text{p}}} \right) \cdot {\upalpha }_{h} \cdot C_{0}$$

16 $$\frac{{dC_{l1} }}{dt} = {\text{p}} \cdot {\upalpha }_{l} \cdot C_{0} - \gamma \cdot C_{l1}$$

17 $$\frac{{dC_{h1} }}{dt} = \left( {1 - {\text{p}}} \right) \cdot {\upalpha }_{h} \cdot C_{0} - \gamma \cdot C_{h1}$$

18 $$\frac{{dS_{l1} }}{dt} = \gamma \cdot C_{l1} - {\upalpha }_{l} \cdot s_{1} \cdot S_{l1}$$

19 $$\frac{{dS_{h1} }}{dt} = \gamma \cdot C_{h1} - {\upalpha }_{h} \cdot s_{1} \cdot S_{h1}$$

20 $$\frac{{dC_{lk} }}{dt} = {\upalpha }_{l} \cdot s_{k - 1} \cdot S_{lk - 1} - \gamma \cdot C_{lk}$$

21 $$\frac{{dC_{hk} }}{dt} = {\upalpha }_{h} \cdot s_{k - 1} \cdot S_{hk - 1} - \gamma \cdot C_{hk}$$

22 $$\frac{{dS_{lk} }}{dt} = \gamma \cdot C_{lk - 1} - {\upalpha }_{l} \cdot s_{k} \cdot S_{lk}$$

23 $$\frac{{dS_{hk} }}{dt} = \gamma \cdot C_{hk - 1} - {\upalpha }_{h} \cdot s_{k} \cdot S_{hk}$$

24 $$\frac{{dC_{0} }}{dt} = - \left( {{\text{r}}_{t} \cdot {\upalpha }_{r} + \left( {1 - {\text{r}}_{t} } \right) \cdot {\upalpha }_{d} } \right) \cdot C_{0}$$

25 $$\frac{{dC_{1} }}{dt} = \left( {{\text{r}}_{t} \cdot {\upalpha }_{r} + \left( {1 - {\text{r}}_{t} } \right) \cdot {\upalpha }_{d} } \right) \cdot C_{0} - \gamma \cdot C_{1}$$

26 $$\frac{{dS_{1} }}{dt} = \gamma \cdot C_{1} - \left( {{\text{r}}_{t} \cdot {\upalpha }_{r} + \left( {1 - {\text{r}}_{t} } \right) \cdot {\upalpha }_{d} } \right) \cdot s_{1} \cdot S_{1}$$

27 $$\frac{{dC_{k} }}{dt} = \left( {{\text{r}}_{t} \cdot {\upalpha }_{r} + \left( {1 - {\text{r}}_{t} } \right) \cdot {\upalpha }_{d} } \right) \cdot s_{k - 1} \cdot S_{k - 1} - \gamma \cdot C_{k}$$

28 $$\frac{{dS_{k} }}{dt} = \gamma \cdot C_{k} - \left( {{\text{r}}_{t} \cdot {\upalpha }_{r} + \left( {1 - {\text{r}}_{t} } \right) \cdot {\upalpha }_{d} } \right) \cdot s_{k} \cdot S_{k}$$

Models are parameterized on the basis of an unsystematic literature review and expert discussions (Table 1). The IRs, which indicate the level of disease spread, are inspired by data published in the R21/Matrix-M vaccine trial [3] and are used to illustrate scenarios of standard (low IRs) and seasonal (higher IRs) transmission sites. To isolate the effects of model structure from parameter magnitude and ensure comparability across models, the IR parameters of each model were calibrated to maintain an average population-specific value of 1.00. Therefore, any differences observed in the models’ outputs are due to their structural differences. In Models A and B all participants have a daily infection risk of α = 1.00/365, which equals an average of 1.00 infections per person per year. This value represents areas with perennial malaria transmission. Model C incorporates the protective effect of vaccination. The effect of the R21/Matrix-M vaccine on recurrent events was considered, with vaccine efficacy reported at 67% for standard sites [3], leading to *v* of 1–0.67 = 0.33. Model D shows heterogeneous transmission and 90% percent of the modeled population are exposed to a lower IR of α_l_ = 0.50/365 and 10% to a higher IR of α_h_ = 5.50/365. Model E represents seasonal transmission with higher IR during the rainy season ranging from October to March (α_r_ = 1.50/365) and a lower rate (α_d_ = 0.50/365) during the other months. In Models B to E, cases are protected from reinfection for 21 days due to assumed treatment [14], and each experienced infection reduces susceptibility to subsequent infection by 10%, with 50% being the lowest possible value. These parameters are not clearly supported by the literature despite a wide range of publications on the subject [15–17], however, these are relatively conservative, biologically plausible values that best enable an overall understanding of the model dynamics. All models are designed with 6 infection compartments (*k* = 6), and results are summarized up to the category of ≥ 4 recurrent infections.

**Table 1:** Parameter settings for the five infection cohort models

Parameter	Model A	Model B	Model C	Model D	Model E	Ref
α (daily IR)	1.00/365	1.00/365	1.00/365	–	–	[3]
*v* (reduced risk due to vaccination)			0.33	0.33		[3]
*p* (proportion high transmission)	–	–		0.1	–	–
α_l_ (daily IR, low transmission)	–	–	–	0.50/365	–	[3]
α_h_ (daily IR, high transmission)	–	–	–	5.50/365	–	[3]
α_d_ (daily IR, dray season)	–	–	–	–	0.50/365	[3]
α_r_ (daily IR, rainy season)	–	–	–	–	1.50/365	[3]
γ (protection rate due to treatment)	–	21 days	21 days	21 days	21 days	[14]
Incremental reduced susceptibility after infection (*s*_*k*_)	–	− 10%	− 10%	− 10%	− 10%	[15–17]
Month of the rainy season (*r*_t_ = 1)					Oct.–Mar	[3]

IR, incidence rate

A sensitivity analysis was conducted using a one-at-a-time approach to show the relative importance of each parameter on the model output. In this approach, each parameter was systematically varied across a predefined range while the other remained unchanged. Deviations in the modelled proportions of cases from the respective baseline models (i.e., models with original parameter settings) using results from the end of year 2 were calculated. The absolute differences in proportions of cases with one, up to two, up to three and up to four infections were reported. The following parameter values were used in the sensitivity analysis:

- of 0.9–1.1 by increments of 0.1;
- *v* of ± 10% by increments of 1%;
- *p* of 5–15% by increments of 1%;
- *tp* of 11–31 days by increments of 1 day;
- *s*_k_ of 0–20% susceptibility reduction after infection by increments of 1%;
- *r*_t_ of − 56–56 days added to the rainy season by increments of 7 days.

Since changing *p* (Model D) and *r*_t_ (Model E) also affects the IR in the modelled populations, the corresponding α values were weighted to maintain IR equal to 1 when *p* or *r*_t_ was varied. In Equation 29, α′_h_ (the weighted daily IR in high-transmission settings used in the sensitivity analysis) were calculated by multiplying α_h_ with the ratio of *p* to *p*_s_, where *p*_s_ is the proportion of the population in high-transmission settings used in the sensitivity analyses. Equation 30 shows the calculation of α′_l_ using the complement of *p* (1–*p*). The same weighting principle was applied for the sensitivity analysis of Model E (Eqs. 31 and 32). The weighted daily IR during the dry season (α′_d_) and rainy season (α′_r_) were calculated using the baseline proportion of rainy days per year (*w*) and the proportion of rainy days in the sensitivity analysis (*w*_s_).

29 $$\alpha{\prime}_{h} = {\upalpha }_{h} \cdot \frac{p}{{p_{s} }}$$

30 $$\alpha{\prime}_{l} = {\upalpha }_{l} \cdot \frac{{\left( {1 - p} \right)}}{{\left( {1 - p_{s} } \right)}}$$

31 $$\alpha{\prime}_{d} = {\upalpha }_{d} \cdot \frac{w}{{w_{s} }}$$

32 $$\alpha{\prime}_{r} = {\upalpha }_{r} \cdot \frac{{\left( {1 - w} \right)}}{{\left( {1 - w_{s} } \right)}}$$

In the last section of the results, a simple example is provided, demonstrating how to estimate final sample size requirements from assumed proportions by dividing the needed case numbers by the respective expected proportion. Furthermore, the expected number of recurrent events in a study cohort is estimated by multiplying the calculated proportions by a given sample size. The models were calculated in *R* (version 4.5.0) using the *deSolve* package (version 1.40) to solve differential equations. The *R*-script is provided in Supplementary File 1.

## Results

**Fig. 2:**
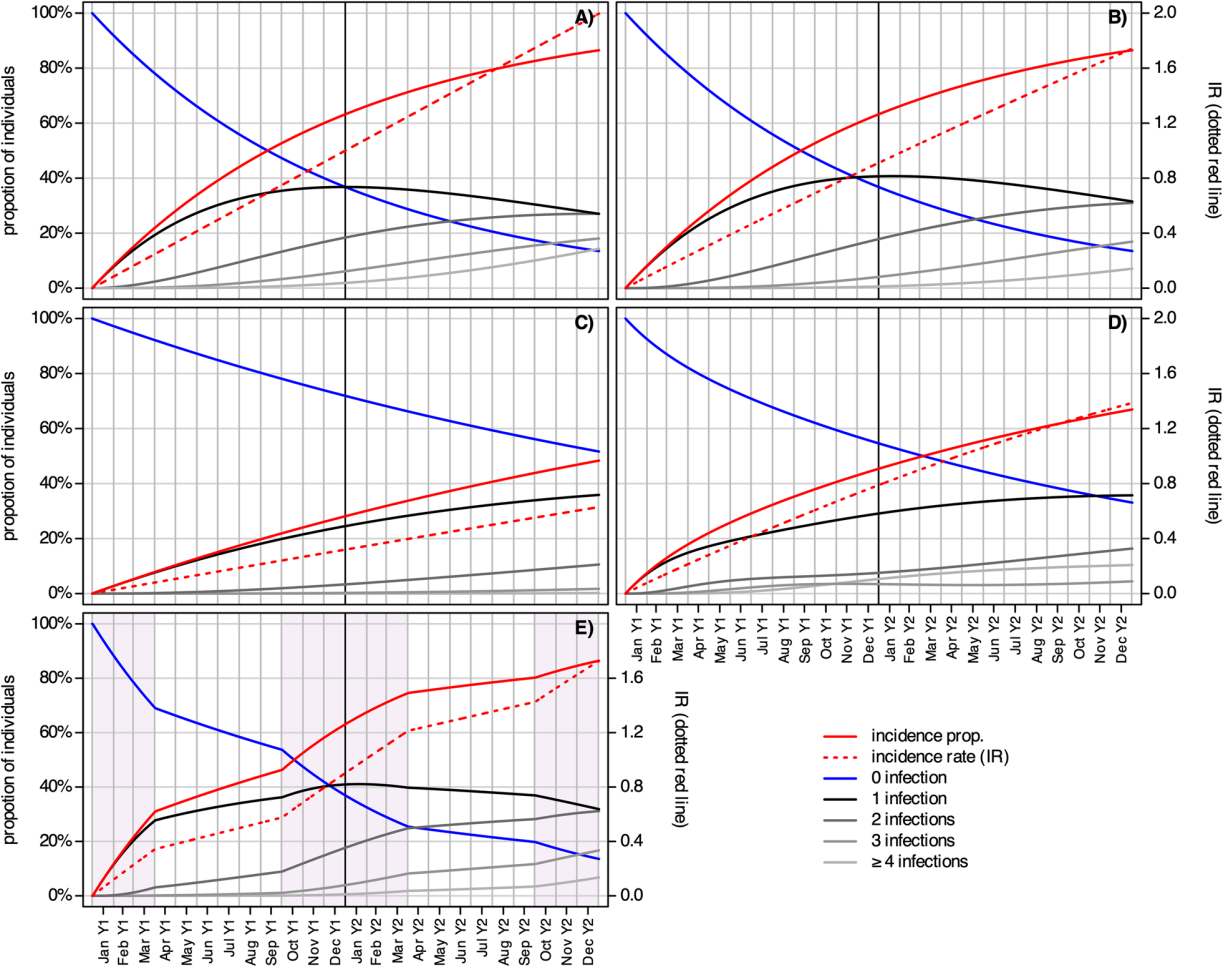
Summary of the three model results: **A** infections occur randomly in an equally susceptible study cohort; **B** infections reduce susceptibility to reinfection, and cases are protected during treatment; **C** as Model B, with infection risk reduced by vaccination; **D** as Model B with heterogeneous infection risk; **E** as in Model B, with seasonal transmission. The shaded areas in E indicate months with high transmission

Model A represents the independent occurrence of recurrent malaria infections over 2 years, considering a constant annual IR of 1.00. Figure 2a shows a steady increase in infections over time, with 63% of the population at 12 months and 86% at 24 months experiencing at least one malaria infection (IP column in Table 2). Over time, individuals who experienced a first infection could become reinfected, and as the number of repeated infections increased (grey shaded lines in Fig. 2a), the increase in single infections slowed (black line in Fig. 2a). At 24 months, 27% experienced one, 27% experienced two, 18% experienced three, and 14% experienced four or more infections. The percentage of individuals who experienced repeated infections (i.e., > 1 infection) was 26% at 12 months and this increased to 59% at 24 months. The dotted red line in Fig. 2a shows the rate of events (i.e., the IR), which was 1.00 at month 12 and 2.00 at month 24, representing the IR used to calculate the model. As the independent occurrence of infections was modelled, the proportion of cases with at least one infection, represented by IP (IP column in Table 2), can also be estimated from the IR by 1-exp(− 1.00) = 63% for 1 year and 1-exp(− 1.00∙2) = 86% for 2 years. As outlined in the Methods section, the results from Model A can also be calculated using the Poisson distribution function (Eq. 4).

**Table 2:** Summary of Model A to D outcomes at 12 and 24 months

Model	0 inf. (%)	1 inf. (%)	2 inf. (%)	3 inf. (%)	≥ 4 inf. (%)	IP (%)	IR
A (12 months)	37	37	18	6	2	63	1.00
A (24 months)	14	27	27	18	14	86	2.00
B (12 months)	37	41	18	4	1	63	0.91
B (24 months)	14	32	31	17	7	86	1.74
C (12 months)	72	25	3	0	0	28	0.32
C (24 months)	52	36	11	2	0	48	0.63
D (12 months)	55	29	8	3	5	45	0.79
D (24 months)	33	36	16	4	10	67	1.39
E (12 months)	37	41	18	4	1	63	0.90
E (24 months)	14	32	31	17	7	86	1.73

*inf.* infection(s); *IP* incidence proportion; *IR* incidence rate

Numbers represent percentages of the modelled study population/cohort belonging to that group and the estimated incidence rates

Model B included the temporal protective effects of treatment and reduced susceptibility after infection (Fig. 2b). The IR of malaria infections was 0.91 at 12 months and 1.74 at 24 months, slightly lower than in Model A. In addition, the proportion of cases who experienced recurrent infections was lower (23% at 12 months and 55% at 24 months). However, the number of cases with at least one infection (as defined by IP) was the same as that in Model A because the occurrence of first infections was not affected by immunity or treatment (Table 2). This demonstrates how treatment and acquired immunity can affect the pattern of recurrent infections even when the initial infection risk is maintained.

Model C uses the same model structure and parameter settings as Model B, but includes the preventive effect of a vaccine (Table 2 and Fig. 2c). A vaccine efficacy of 67% reduces the IR to 0.32 and 0.63 by the end of years one and two, respectively. Recurrent events were also observed less frequently, with 36, 11, 2 and 0% of individuals experiencing one, two, three or four or more infections by the end of year two, respectively.

Model D shows the effect of heterogeneous transmission (Fig. 2d). After 1 year 45% and after 2 years 67% of the cohort experienced malaria infections with an IR of 0.79 and 1.39, respectively (Table 2). Recurrent infections were observed in 16% of the total cohort at 1 year and in 31% at 2 years. However, after 2 years, nearly all individuals in the high transmission group experienced more than one infection (100%), compared to 24% in the low transmission group.

Model E shows seasonal transmission. The red lines in Figure E clearly show the strongest increase in cases during the rainy season (gray shaded areas). After 1 year, 63% of the cohort experienced malaria infections with an IR of 0.90 at 1 year, and after 2 years, 86% experienced malaria infections with an IR of 1.73 infections at 2 years. The proportion of individuals with recurrent infections was 22% at 1 year and 55% at 2 years.

**Fig. 3:**
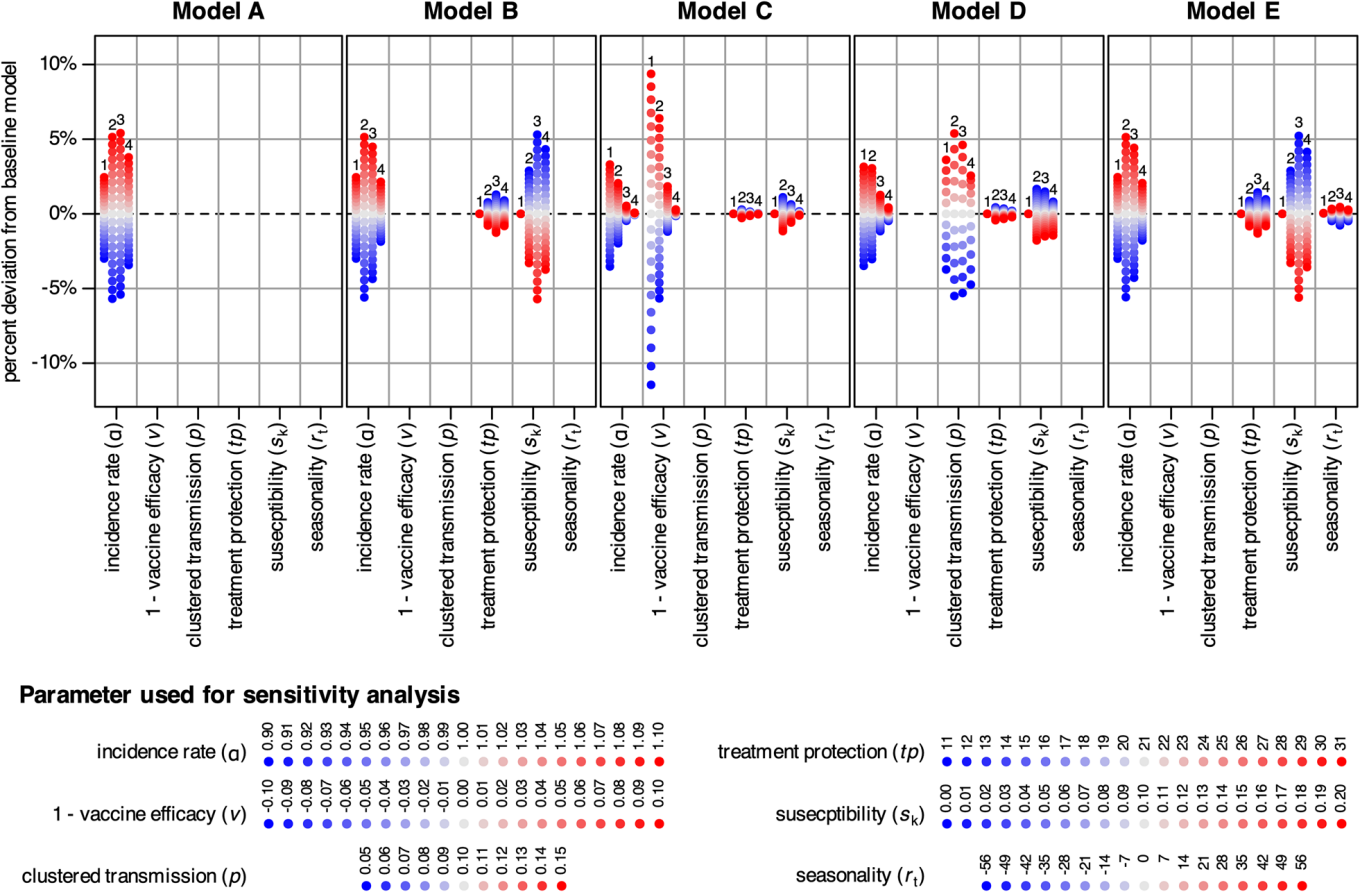
Summary of the sensitivity analysis (percentage change from the baseline model) for all the parameters used in the respective models. The numbers in the plot area indicate results for one (1), up to two (2), up to three (3) and up to four (4) infections

The sensitivity analysis (Fig. 3) revealed distinct parameter importance across the five models. The transmission rate (α) showed a strong influence in Models A, B, and E, where increasing α by 10% raised the number of recurrent infections (≥ 2 infections) by approximately 5%, while decreasing α by 10% reduced recurrent infections by about 6%. This effect was considerably smaller in Models C and D, where other parameters played a stronger role. In Model C, changes in vaccine efficacy (*v*) had the strongest effect and reducing *v* by 10% decreased the proportion of recurrent infections by 5.7%, whereas increasing *v* by 10% increased it by 6.4%. In Model D, clustered transmission (*p*) dominated the outcome. Setting *p* to 5% lowered recurrent infections by 5.5%, while increasing *p* to 15% raised them by 5.4%. Susceptibility reduction after infection (*s*_k_) had the greatest impact in Models B and E, especially when considering ≥ 3 recurrent infections. Removing susceptibility effects (*s*_k_ = 0) increased the proportion of recurrent infections by about 5%, whereas decreasing susceptibility after infection to 20% reduced it by approximately 6%. In Models C and D, susceptibility played only a minor role. Finally, treatment protection (*tp*) and seasonality (*r*_t_) showed only small effects on the estimated proportion of recurrent infections in all models.

To estimate the total sample size of a cohort, assumptions need to be made about the required number of cases and divided by the respective proportions calculated in the model. For example, if sample size estimations show that 50 participants with recurrent infections are required, this number must be divided by the modelled expected proportion of participants with recurrent events (i.e., ≥ 2 infections). Using the numbers from Model A–E above, and considering a 2-year observation period, cohorts of 50/23% = 217 participants in Model A, 50/20% = 250 in Model B, 50/3% = 1667 in Model C, 50/28% = 179 in Model D, and 50/65% = 77 in Model E are needed. These calculations demonstrate how different assumptions about disease dynamics can lead to substantially different sample size requirements, ranging from 77 to 1667 participants for the same number of required cases (i.e., n = 50). Finally, assuming that cohorts of 500 individuals are followed for 2 years, estimates from the calculated models lead to an expected number of individuals with recurrent events of 115 for Model A (500 × 23%), 100 for Model B (500 × 20%), 15 for Model C (500 × 3%), 140 for Model D (500 × 28%), and 325 for model E (500 × 65%).

## Discussion

Recurrent events in longitudinal studies cannot be directly derived from disease frequency data. The occurrence of events is a dynamic process, and recurrent infections occur over time within growing groups of cases with previous infections [10]. This study provides a simple set of differential equations to estimate the number of recurrent events in malaria study cohorts which can be adjusted to represent other study settings. The five models demonstrate how the assumption of disease dynamics can substantially impact the expected pattern of recurrent infections and consequently influence sample size considerations.

Model A represents the expected disease dynamics if the event risk is constant within a population and the level of immunity equals among individuals. This would be the case for respiratory infections like influenza, in which immunity wanes quickly, and infected individuals gain susceptibility very slowly or where mutations in the viral genome result in viruses that evade immunity [18]. The example of malaria is used in our models, where naïve individuals lose susceptibility to (symptomatic) infection after experiencing (repeated) infections. Model B demonstrates such an effect of increasing immunity. This model represents a cohort of immune-naïve infants in a vaccination trial, in which all infants were fully susceptible at the beginning of the study without any previous malaria infection. Model B shows the same IP as Model A because first infections are not prevented by immunity effects. However, the calculated IR was reduced due to increasing immunity and treatment effects over time. The development of immunity is a complex process, and various aspects have been discussed in the literature. For example, for malaria, even one to two infections in young children can provide immune protection against severe malaria [19]. However, immunity against clinical episodes in general is more complex. Some studies suggest that clinical immunity can be achieved over 10 years in areas of medium–high transmission [16, 20]; this immune development may occur even faster in higher transmission areas. Thus, in low-transmission areas, immune development can take significantly longer or not develop at all, as immune protection gained from one infection may wane prior to a subsequent infection. For Models B–E, we selected a 10% reduction in susceptibility after each infection episode and ignored the effect of waning immunity or time-intervals between infections. However, as mentioned in the methods, these parameters are assumptions not directly support by the literature. Model C is very similar to Model B, but it considers the preventive effect of a vaccination intervention. It mimics the occurrence of recurrent events in an intervention group within a clinical cohort.

A further extension is heterogeneous disease occurrence. Model D illustrates disease dynamics in which a subgroup of the cohort is highly exposed to infection. The overall number of individuals with recurrent infections was 28%; however, nearly 100% of individuals in the high-transmission group experienced more than one infection. Diseases like malaria are known to vary over even small geographical areas [21, 22]. In addition, differences in individual risk of infection are described [17, 23, 24], which supports the idea of modelling heterogeneous disease transmission to plan study cohorts. Seasonality, as considered in Model E, is another factor which has a strong effect on disease dynamics. In particular, if sample size considerations should also inform study conduct, like availability of laboratory resources for diagnostics or personnel needed to collect samples over time, these temporal variations provide valuable information for study implementation.

Models B to E take into account the impact of immunity and begin with a malaria-naïve population. In this scenario, infants with maternal antibodies are recruited into a clinical cohort and experience their first infections during the study. However, when considering an adult population that may already have experienced several malaria infections, susceptibility can be ignored because the cohort may already have partial immunity. Therefore, extensions of Model A that ignore decreasing susceptibility may be sufficient. However, cohorts with mixed susceptibility groups (i.e., different age groups) require stratified models, in which the fractions of participants have different levels of susceptibility.

The compartmental models and their associated differential equations can be adapted to represent a greater number of recurrent infections (*C*_*k*_). In settings with a high incidence rate, where a greater number of infections per person are expected, the models should be extended to appropriately represent these dynamics. While there is no formal rule for selecting the optimal number of recurrent infection compartments, *k* should be chosen to adequately capture the number of recurrent infections required to answer the research question. It is recommended that at least two additional sets of recurrent infections are incorporated into the equations, to properly characterise the dynamics of the extended reinfection cycles. This approach provides flexibility to add further infection compartments if recurrence is not well represented in the established model. This ensures that model simplification does not artificially restrict the observed infection dynamics.

When models are parameterized, it must be considered that modelling protective effects (e.g., treatment or reduced susceptibility after infection) will reduce the calculated IR compared to the IR used as a model parameter for transmission intensity. The input IR, however, represents the overall risk of infection in a given study area, which should be represented in the modelled population. Hence, to parameterize a model, the input IR could be increased until the model output yields the IR or IP as expected in the study population.

The applied differential equations generate deterministic results without confidence intervals, and a parameterized model always yields the same numbers. However, the occurrence of infections is influenced by chance, and different outcomes are plausible under the same assumptions. In addition, fixed model parameters were used; however, probability distributions provide a more realistic representation of several parameters. Stochastic models can account for these assumptions and generate a range of possible model outputs that exhibit random effects on the modeled results [25]. In particular, if cohorts of a small number of individuals are planned or infections occur at a low rate, random effects must be considered. As described above, finding appropriate model parameters can be challenging, and various parameter assumptions can often be justified from the literature. For example, local IRs for malaria based on incomplete surveillance data can vary significantly. The sensitivity analysis also underscores the importance of this point, showing the strong effects of applied parameter values, such as the incidence rate (α), vaccine efficacy (*v*), clustered transmission (*p*) and susceptibility after infection (*s*_k_), on model outcomes. Therefore, when constructing or parameterizing a mathematical model, careful consideration must be given to data validity and parameter uncertainty. Well-founded parameter estimates are essential for reliable model outcomes and meaningful interpretation. The presented models have different levels of complexity, which must be considered when interpreting the results. For example, Model A assumes constant susceptibility among all individuals, which ignores the effect of immunity after a previous infection, whereas Model B’s and C’s incremental reduction in susceptibility after infection simplifies complex immunological processes that may vary between individuals and transmission settings. Model D’s binary transmission risk does not fully capture the spectrum of exposure heterogeneity or risk differences over time. Finally, Model E, which incorporates temporality, assumes stable seasonal IRs that are likely to vary over time and between years. These limitations highlight the importance of carefully selecting and potentially adapting these models to specific study contexts and local epidemiological patterns. However, adding complexity always requires additional information, which increases model uncertainty. Thus, the model’s complexity must be balanced against the available evidence to inform the calculations.

## Conclusion

Accurate translation of the required case numbers into recruitment targets is crucial for successful study planning and resource allocation. The models presented here provide a structured approach to this challenge, particularly for studies involving recurrent events. The five presented models were calculated with different levels of complexity, and the underlying equations can be easily adjusted to represent further patterns of disease occurrence. As highlighted above, a proper understanding of the study population and the underlying disease dynamics is necessary to construct a reasonable model and to find an appropriate parameter setting. These model equations provide a simple solution to support sample size considerations in longitudinal studies. Moreover, these models allow researchers to better anticipate the study duration required to observe sufficient recurrent events, thus balancing study costs and scientific objectives.

## Supplementary Information

Additional file 1.

## Data Availability

The *R* script to calculate the mathematical models is available as Supplementary File 1.
